# MiMultiCat: A Unified Cloud Platform for the Analysis of Microbiome Data with Multi-Categorical Responses

**DOI:** 10.3390/bioengineering11010060

**Published:** 2024-01-08

**Authors:** Jihun Kim, Hyojung Jang, Hyunwook Koh

**Affiliations:** Department of Applied Mathematics and Statistics, The State University of New York (SUNY), Incheon 21985, Republic of Korea

**Keywords:** microbiome data analysis, cloud computing, human microbiome, multi-categorical response, microbiome association testing, microbiome prediction modeling

## Abstract

The field of the human microbiome is rapidly growing due to the recent advances in high-throughput sequencing technologies. Meanwhile, there have also been many new analytic pipelines, methods and/or tools developed for microbiome data preprocessing and analytics. They are usually focused on microbiome data with continuous (e.g., body mass index) or binary responses (e.g., diseased vs. healthy), yet multi-categorical responses that have more than two categories are also common in reality. In this paper, we introduce a new unified cloud platform, named MiMultiCat, for the analysis of microbiome data with multi-categorical responses. The two main distinguishing features of MiMultiCat are as follows: First, MiMultiCat streamlines a long sequence of microbiome data preprocessing and analytic procedures on user-friendly web interfaces; as such, it is easy to use for many people in various disciplines (e.g., biology, medicine, public health). Second, MiMultiCat performs both association testing and prediction modeling extensively. For association testing, MiMultiCat handles both ecological (e.g., alpha and beta diversity) and taxonomical (e.g., phylum, class, order, family, genus, species) contexts through covariate-adjusted or unadjusted analysis. For prediction modeling, MiMultiCat employs the random forest and gradient boosting algorithms that are well suited to microbiome data while providing nice visual interpretations. We demonstrate its use through the reanalysis of gut microbiome data on obesity with body mass index categories. MiMultiCat is freely available on our web server.

## 1. Introduction

The field of the human microbiome is rapidly growing due to the recent advances in high-throughput sequencing technologies (e.g., 16S rRNA amplicon sequencing [1,2] and shotgun metagenomics [3]). Investigators currently seek to discover novel biomarkers that are crucial to human health or disease (e.g., cancer [4,5,6], diabetes [7,8], obesity [9,10], intestinal disease [11,12], oral disease [13], nasal disease [14]) through the human microbiome.

There have also been many new analytic pipelines, methods and/or tools developed for microbiome data preprocessing and analytics [15,16,17,18,19,20,21,22,23,24,25,26,27,28,29,30,31]. They are usually focused on the microbiome data with continuous (e.g., body mass index) or binary responses (e.g., diseased vs. healthy). However, in reality, multi-categorical responses that have more than two categories are also common. Multi-categorical responses in human microbiome studies are, for example, (i) stages I, II, III and IV for cancer/diabetes, (ii) underweight, normal, overweight and obese for obesity, (iii) extremely preterm, very preterm and moderate to late preterm for preterm birth and so forth. Nevertheless, currently, there is no well-planned routine software for the analysis of microbiome data with multi-categorical responses.

In this paper, we introduce a new unified cloud platform, named MiMultiCat, for the analysis of microbiome data with multi-categorical responses. The two main distinguishing features of MiMultiCat that we stress are as follows: First, MiMultiCat streamlines a long sequence of microbiome data preprocessing and analytic procedures extensively on user-friendly web interfaces. Microbiome data are highly complex, requiring a broad spectrum of expertise to deal with them. It is also time-consuming and laborious to write computer codes on command-line interfaces. Yet, MiMultiCat resolves all such hassles based on ‘easy-to-use’ and ‘step-by-step’ web environments and, thus, can benefit many people in various disciplines (e.g., biology, medicine, public health). Second, MiMultiCat performs both association testing and prediction modeling extensively. For association testing, MiMultiCat handles both ecological (e.g., alpha and beta diversity) and taxonomical (e.g., phylum, class, order, family, genus, species) contexts through covariate-adjusted or unadjusted analysis. Investigators seek not only to comprehend the microbiome as a microbial community or ecosystem but also seek to dissect it into upper- and lower-level microbial taxa. Covariate-adjusted analytics are also necessary to prevent spurious discoveries especially for observational studies, in which many potential confounding factors (e.g., age, sex) can be involved. Here, we emphasize that MiMultiCat is designed to satisfy such analytic demands extensively. For prediction modeling, MiMultiCat employs the random forest [32] and gradient boosting [33,34] algorithms that are well suited to microbiome data to account for possibly highly non-linear patterns of relationships. They are also easy to understand and interpret while providing nice visual representations to rank microbial taxa in importance and illustrate the delicate patterns of the relationships to human health or disease responses.

We organize the rest of the paper as follows: In the following Materials and Methods section, we describe the underlying statistical methods, web server architecture and example data for MiMultiCat. Then, in the Results section, we describe all the modules of MiMultiCat along with its applications to gut microbiome data on obesity with body mass index (BMI) categories [35]. Finally, in the Discussion section, we summarize and finish with concluding remarks.

## 2. Materials and Methods

### 2.1. Association Testing

Alpha diversity and taxonomic analysis (Table 1): For a nominal response variable, MiMultiCat employs the multinomial logistic regression model for covariate-adjusted analysis and (i) the parametric analysis of variance (ANOVA) F-test for global testing and Tukey’s honestly significant difference test for pairwise comparisons [36], (ii) the non-parametric Kruskal–Wallis test [37] for global testing along with Dunn’s test [38] for pairwise comparisons and (iii) multinomial logistic regression for unadjusted analysis. For an ordinal response variable, MiMultiCat employs the proportional odds regression model [39] for both covariate-adjusted and unadjusted analysis.

Beta diversity analysis (Table 2): For a nominal response variable, MiMultiCat employs the microbiome kernel association test for multi-categorical outcomes (MiRKAT-MC) [40] for covariate adjusted analysis and non-parametric multivariate analysis of variance (PERMANOVA) [41,42] and MiRKAT-MC [40] for unadjusted analysis. For an ordinal response variable, MiMultiCat employs MiRKAT-MC [40] for both covariate-adjusted and unadjusted analyses.

### 2.2. Prediction Modeling

For prediction modeling, MiMultiCat employs the random forest [32] and gradient boosting [33,34] algorithms. Microbiome data are highly complex with high dimensionality, sparsity, compositionality and phylogenetic relationships; as such, the patterns of the relationship between microbial taxa and health or disease responses can be highly discrete or irregular. Thus, tree ensemble algorithms, such as the random forest [32] and gradient boosting [33,34] algorithms, can be well suited while robustly accounting for non-linear patterns and decorrelating compositional/phylogenetic relatedness [31].

For the random forest algorithm [32], MiMultiCat trains it to tune the number of randomly selected taxa for each tree in the ensemble through cross-validation based on the loss of Gini impurity. MiMultiCat reports two main outputs for the random forest [32] using (i) a variable importance plot to rank microbial taxa in classification ability and (ii) a partial dependence plot to display the relationship patterns between microbial taxa and muti-categorical responses.

For the gradient boosting algorithm [33,34], MiMultiCat trains it slowly using a small learning rate with or without $L_{2}$ regularization [34]. MiMultiCat tunes the number of trees (i.e., the number of iterations) and the number of partitions for each tree in the ensemble (also known as the interaction depth) simultaneously through cross-validation based on the loss of cross-entropy. For gradient boosting [33,34], a popular software package, XGBoost [34], was employed for fast C++ computations. MiMultiCat reports two main outputs using (i) a Shapley additive explanation (SHAP) plot to rank microbial taxa in classification ability and (ii) a partial dependence plot to display the relationship patterns between microbial taxa and multi-categorical responses.

### 2.3. Web Server Architecture

We wrote MiMultiCat using R language and constructed its app interfaces using R shiny 1.7.5. We deployed it onto the web (http://mimulticat.micloud.kr, access date: 26 December 2023) using ShinyProxy 2.6.1 (https://www.shinyproxy.io, access date: 1 November 2023) and Apache2 (https://httpd.apache.org, access date: 1 November 2023). Our web server runs on the operating system Ubuntu 20.04 (https://ubuntu.com, access date: 1 November 2023) and the computing device ThinkCentre Neo 50S Gen3 (Lenovo, Quarry Bay, Hong Kong) that comes with the specifications of an Intel Core i9-12900 (16-core) processor (Intel, Santa Clara, CA, USA) and 64 GB DDR4 memory (Samsung, Seoul, Republic of Korea). MiMultiCat can accommodate up to ten concurrent web server users. When the server is busy, users can alternatively run it using their local computer through our GitHub repository (https://github.com/jkim209/mimulticatgit, access date: 26 December 2023).

### 2.4. Data Availability

We describe each module in the following Results section using public gut microbiome data on obesity (see Application notes) published in [35], where BMI was used as a measure of obesity. We categorized it into three levels, normal (18.5 ≤ BMI < 25), overweight (25 ≤ BMI < 30) and obese (30 ≤ BMI). The 16S raw sequence data are publicly available from the European Bioinformatics Institute (EMBL-EBI) database with access numbers ERP006339 and ERP006342 [35]. We processed them using QIIME 1.7.0 [15,16] and FastTree [43] based on the GreenGenes 12.10 database (https://greengenes.secondgenome.com, access date: 1 November 2023) to construct the feature table, taxonomic table and phylogenetic tree. The final processed microbiome data together with the meta/sample data are also available as example data in the Data Input module so that our users can easily comprehend suitable data formats.

## 3. Results

### 3.1. Data Processing

The *Data Processing* module is composed of three sub-modules, *Data Input*, *Quality Control* and *Data Transformation* as in [29,30,31].

First, in the *Data Input* module, users can upload microbiome data using a unified format, called phyloseq [25], or using separate files. Then, in the *Quality Control* module, they can perform quality controls with respect to (i) the kingdom of interest (default: Bacteria); (ii) minimum library size (i.e., total read count) to remove the individuals that have low library sizes (default: 3000); (iii) minimum mean proportion (i.e., relative abundance) to remove the features (i.e., operational taxonomic units (OTUs) or amplicon sequence variants (ASVs)) that have low mean proportions (default: 0.002%); and (iv) errors in taxonomic names to be removed from the taxonomic table. MiMultiCat (i) shows the sample size and the numbers of features, phyla, classes, orders, families, genera and species and (ii) creates interactive histograms and box plots for the library sizes across individuals and the mean proportions across features. Then, finally, in the *Data Transformation* module, MiMultiCat computes ecological indices (i.e., nine alpha diversity indices: Observed, Shannon [44], Simpson [45], Inverse Simpson [45], Fisher [46], Chao1 [47], abundance-based coverage estimator (ACE) [48], incidence-based coverage estimator (ICE) [49] and phylogenetic diversity (PD) [50] and five beta-diversity indices: Jaccard dissimilarity [51], Bray–Curtis dissimilarity [52], Unweighted UniFrac distance [53], Generalized UniFrac distance [54], Weighted UniFrac distance [55]). MiMultiCat also normalizes taxonomic abundances using the widely used methods of centered-log ratio (CLR) [56], rarefaction [57], proportion and arcsine root transformation.

Application notes: First, we uploaded the example gut microbiome data on obesity through the *Data Input* module. Second, we applied the default quality control settings, and then rescued 968 individuals for 484 features (7 phyla, 15 classes, 20 orders, 29 families, 44 genera, 29 species) through the *Quality Control* module. Finally, we computed ecological indices and normalized taxonomic abundances through the *Data Transformation* module.

### 3.2. Data Analysis: Association

The purpose of the *Data Analysis: Association* module is to conduct microbiome association testing with respect to ecological (e.g., alpha and beta diversity) and taxonomical (e.g., phylum, class, order, family, genus, species) contexts; as such, it is composed of three sub-modules, *Alpha Diversity*, *Beta Diversity* and *Taxonomic Analysis*. For this, users need to select a multi-categorical response variable and set up its variable type as nominal or ordinal. Then, they can reorder/rename the categories of the selected response variable. This is to set up the reference category, to change the orders of the categories and/or to change the names of the categories to be better displayed on the output plots. Then, users need to select covariates (e.g., age, sex) for covariate-adjusted analysis or not for unadjusted analysis. Then, they are supposed to select a statistical method from the list of available statistical methods as in Table 1 and Table 2.

For taxonomic analysis, users additionally need to select a data format such as CLR [56] (default), proportion, arcsine root or rarefied counts [57] and taxonomic ranks to be surveyed as ‘from phylum to genus (16S)’ or ‘from phylum to species (metagenomics)’. MiMultiCat applies the Benjamini–Hochberg procedures [58] to control for false discovery rate (FDR) per taxonomic rank.

**Figure 1:**
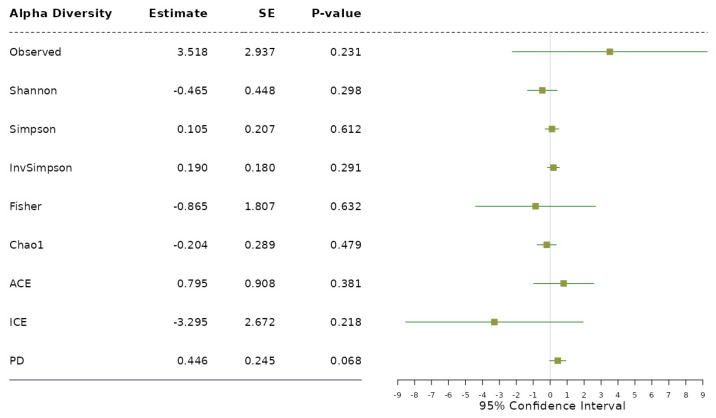
The results from alpha diversity analysis on the association between each alpha diversity index and the levels of BMI adjusted for age and sex based on the proportional odds model.

**Figure 2:**
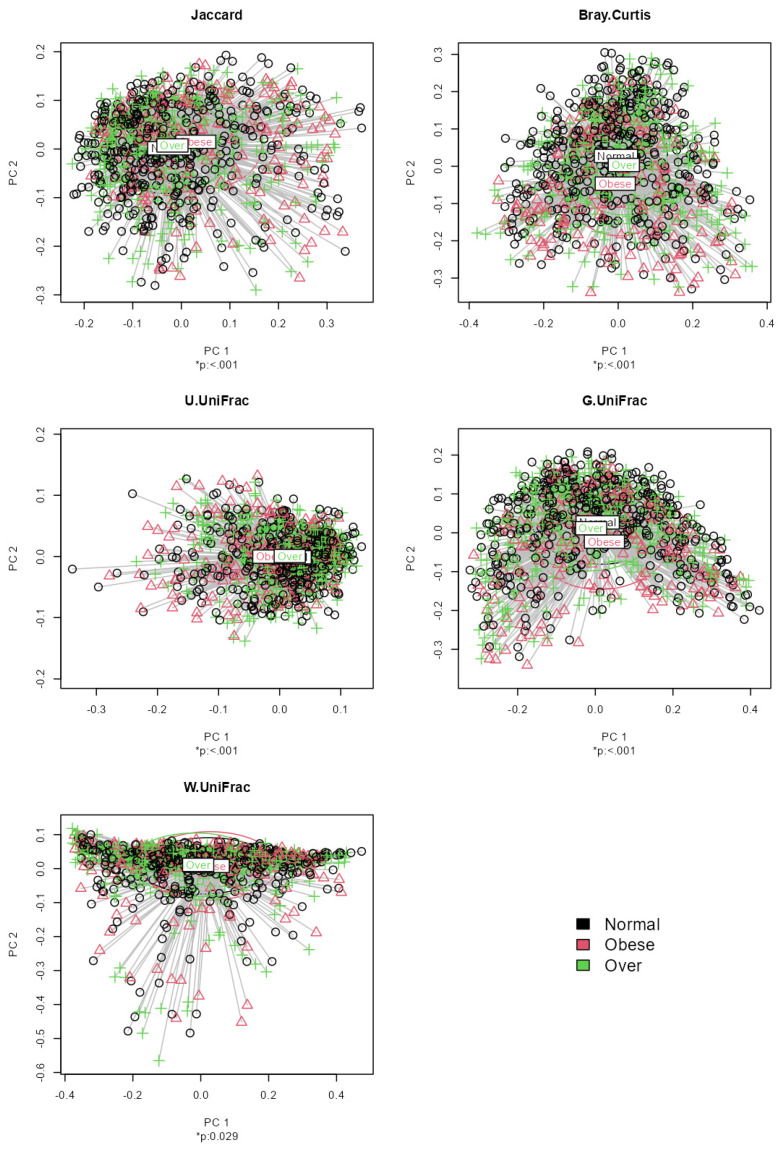
The results from beta diversity analysis on the association between each beta diversity index and the levels of BMI adjusted for age and sex based on MiRKAT-MC.

**Figure 3:**
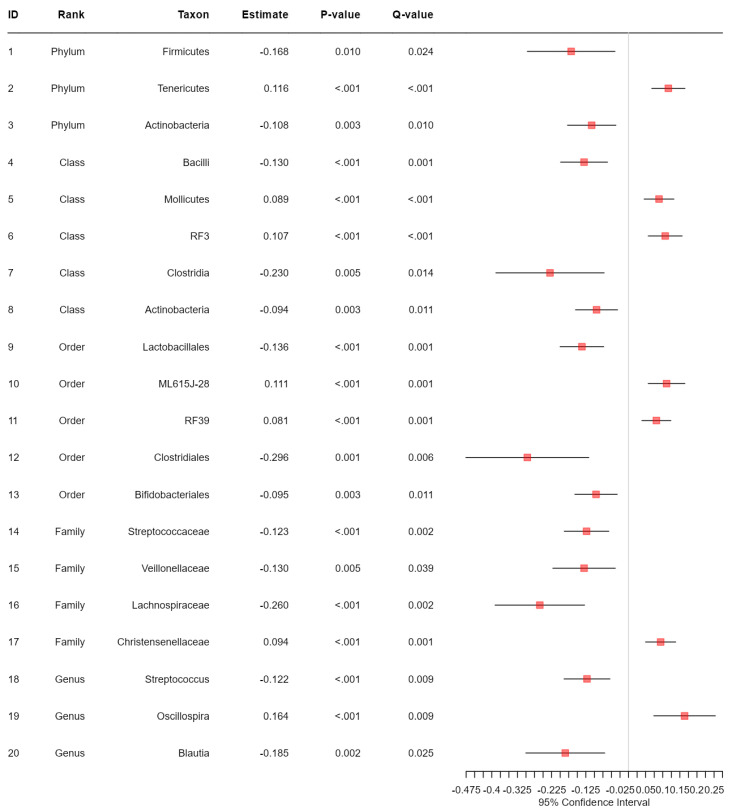
The results from taxonomic analysis on the association between each microbial taxon and the levels of BMI adjusted for age and sex based on the proportional odds model. Q-value represents FDR-adjusted *p*-value.

Application notes: We selected the categorical BMI variable as the response variable and set up its variable type as ordinal. Then, we changed the names of the categories to Normal (level 1), Over (level 2) and Obese (level 3). Then, we selected age and sex as covariates to be adjusted. Then, we selected the proportional odds model [39] for alpha diversity analysis and taxonomic analysis and MiRKAT-MC [40] for beta diversity analysis. For taxonomic analysis, we selected CLR [56] for the data format and ‘from phylum to genus (16S)’ for the taxonomic ranks to be surveyed. Then, we observed no significant association between alpha diversity and the BMI level with respect to any alpha diversity indices at the significance level of 0.05 (Figure 1). However, we observed significant disparities in beta diversity with respect to the Jaccard dissimilarity [51], Bray–Curtis dissimilarity [52], Unweighted UniFrac distance [53], Generalized UniFrac distance [54] and Weighted UniFrac distance [55], across the levels of BMI at the significance level of 0.05 (Figure 2). Finally, we found three phyla (*Fimicutes*, *Tenericutes*, *Actinobacteria*), five classes (*Bacilli*, *Mollicutes*, *RF3*, *Clostridia*, *Actinobacteria*), five orders (*ML615J-28*, *RF39*, *Clostridiales*, *Bifidobacteriales*), four families (*Streptococcaceae*, *Veillonellaceae*, *Lachnospiraceae*, *Christensenellaceae*) and three genera (*Streptococcus*, *Oscillospira*, *Blautia*) to be significantly associated microbial taxa at a false discovery rate of 0.05 (Figure 3).

### 3.3. Data Analysis: Prediction

The purpose of the *Data Analysis: Prediction* module is to conduct microbiome prediction modeling using the random forest [32] and gradient boosting [33,34] algorithms. For this, users first need to select a data format such as CLR [56] (default), proportion, arcsine root or rarefied counts [57]. Then, they need to select a multi-categorical response variable and can reorder/rename the categories of the selected response variable.

For the random forest algorithm [32], users need to select (i) 5-fold or 10-fold cross-validation, (ii) the number of trees to be aggregated in the ensemble (default: 1000), (iii) the maximum number of taxa to be displayed in the variable importance and partial dependence plots (default: 20) and (iv) taxonomic ranks to be surveyed ‘from phylum to genus (16S)’ or ‘from phylum to species (metagenomics)’. Note that we set up the number of trees to be aggregated in the ensemble as 1000 for quick check-ups, but the random forest algorithm [32] has no overfitting issue with an increase in the number of trees. Instead, an increase in the number of trees is only favorable in making the error rate stable with a sufficient convergence [32]. Thus, we recommend increasing the number of trees as much as possible (e.g., 10,000), but this comes with the cost of slow computation.

For the gradient boosting [33,34], users need to select (i) 5-fold or 10-fold cross-validation, (ii) the maximum number of trees in the boosting process (default is 1000), (iii) the learning rate (default: 0.005), (iv) the number of taxa to be displayed in the variable importance and partial dependence plots (default: 20) and (v) taxonomic ranks to be surveyed ‘from phylum to genus (16S)’ or ‘from phylum to species (metagenomics)’. Note that we set up the maximum number of trees in the boosting process as 1000 and the learning rate as 0.005. First, the gradient boosting [33,34] may have an overfitting issue with an increase in the number of trees, but as we described in *Materials and Methods: 2.2 Prediction modelling*, MiMultiCat tunes the number of trees through cross-validation; as such, we can avoid overfitting. We wrote it as the maximum number of trees, not the number of trees; as such, it is only about the capacity of candidate numbers of trees to be tuned. If it is small, the gradient boosting [33,34] can stop early, leading to underfitting. Thus, we recommend increasing the maximum number of trees as much as possible (e.g., 10,000), but this comes with the cost of slow computation. Second, the learning rate is the rate of newly fitted trees reflected in the update [33,34]. If it is small, the tree grows slowly; as such, we can fine-tune it, making the error rate stable with sufficient convergence [33,34]. Thus, we recommend a small learning rate (e.g., 0.001) but, again, this comes with the cost of slow computation.

Note that if any of the above training settings for the random forest [32] or gradient boosting [34] algorithms change, the fitted model will change, leading to different results. This is an issue, but it is not unique to the random forest [32] or gradient boosting [34] algorithms. Indeed, there is no machine learning algorithm that provides closed-form results; as such, the results can vary across different runs. However, since we set up a seed number, MiMultiCat provides the same results under the same training settings; as such, the same result can be reproduced under the same training settings.

Application notes: We selected CLR [56] as the data format and the categorical BMI variable as the response variable. Then, we changed the names of the categories to Normal (level 1), Over (level 2) and Obese (level 3).

**Figure 4:**
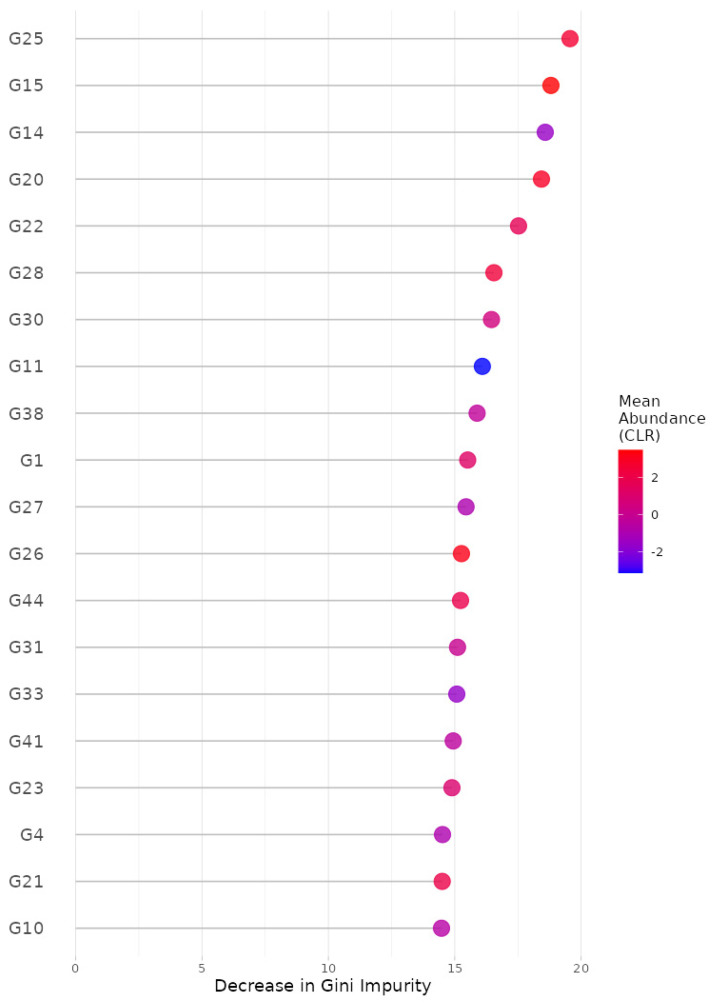
The variance importance at the genus level from random forest algorithm. G25: *Lachnospira*; G15: *Oscillospira*; G14: *SMB53*; G20: *Roseburia*; G22: *Dorea*; G28: *Akkermansia*; G30: *Bilophila*; G11: *Acidaminococcus*; G38: *Butyricimonas*; G1: *Streptococcus*; G27: *Collinsella*; G26: *Bifidobacterium*; G44: *Sutterella*; G31: *Prevotella*; G33: *Paraprevotella*; G41: *Haemophilus*; G23: *Anaerostipes*; G4: *Eubacterium*; G21: *Ruminococcus*; G10: *Phascolarctobacterium*.

**Figure 5:**
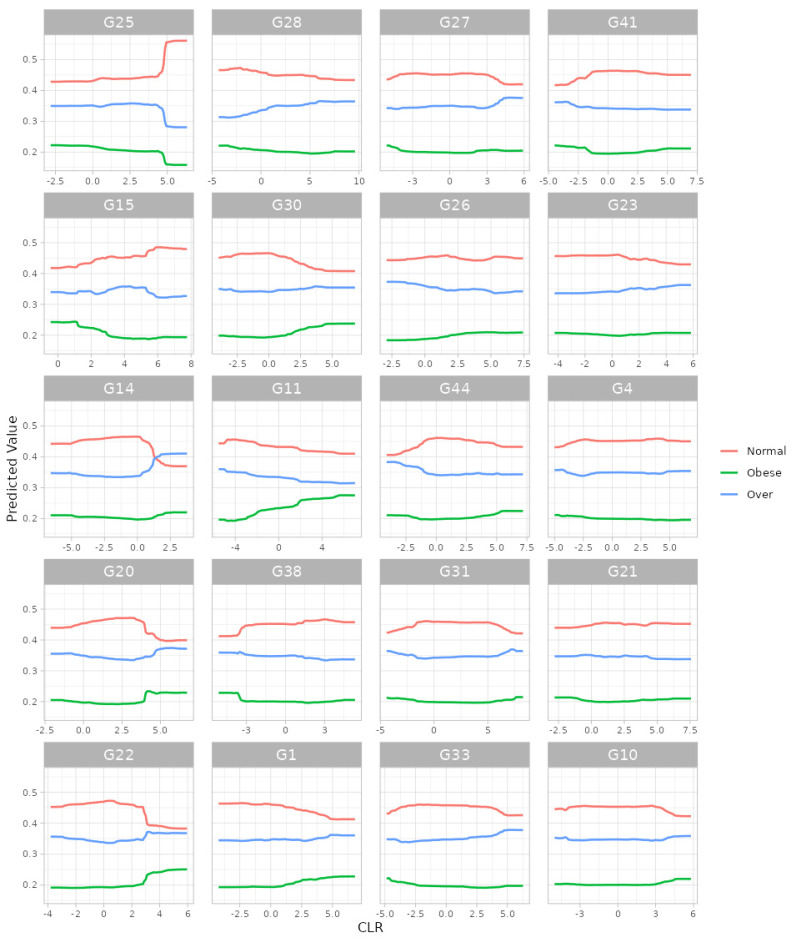
The partial dependence plot at the genus level from random forest algorithm. G25: *Lachnospira*; G15: *Oscillospira*; G14: *SMB53*; G20: *Roseburia*; G22: *Dorea*; G28: *Akkermansia*; G30: *Bilophila*; G11: *Acidaminococcus*; G38: *Butyricimonas*; G1: *Streptococcus*; G27: *Collinsella*; G26: *Bifidobacterium*; G44: *Sutterella*; G31: *Prevotella*; G33: *Paraprevotella*; G41: *Haemophilus*; G23: *Anaerostipes*; G4: *Eubacterium*; G21: *Ruminococcus*; G10: *Phascolarctobacterium*.

For the random forest algorithm [32], we selected (i) 5-fold cross-validation, (ii) the number of trees to be aggregated in the ensemble as 10,000, (iii) the maximum number of taxa to be displayed as 20 and (iv) taxonomic ranks to be surveyed as ‘from phylum to genus (16S)’. Here, we selected the maximum possible number of trees (i.e., 10,000) since we intended to gladly endure some slow computation to obtain better results. Then, we found, at the genus level, G25: *Lachnospira*; G15: *Oscillospira*; G14: *SMB53*; G20: *Roseburia*; G22: *Dorea*; G28: *Akkermansia*; G30: *Bilophila*; G11: *Acidaminococcus*; G38: *Butyricimonas*; G1: *Streptococcus*; G27: *Collinsella*; G26: *Bifidobacterium*; G44: *Sutterella*; G31: *Prevotella*; G33: *Paraprevotella*; G41: *Haemophilus*; G23: *Anaerostipes*; G4: *Eubacterium*; G21: *Ruminococcus*; and G10: *Phascolarctobacterium* as the top 20 most important microbial taxa in predicting levels of BMI (Figure 4). We can also see their relative abundances are larger for lower levels of BMI (Figure 5). Thus, these genera might be beneficial microbes in preventing obesity. This may indicate in a clinical sense that we can lower obesity levels by increasing their relative abundances using dietary supplements or therapeutics.

**Figure 6:**
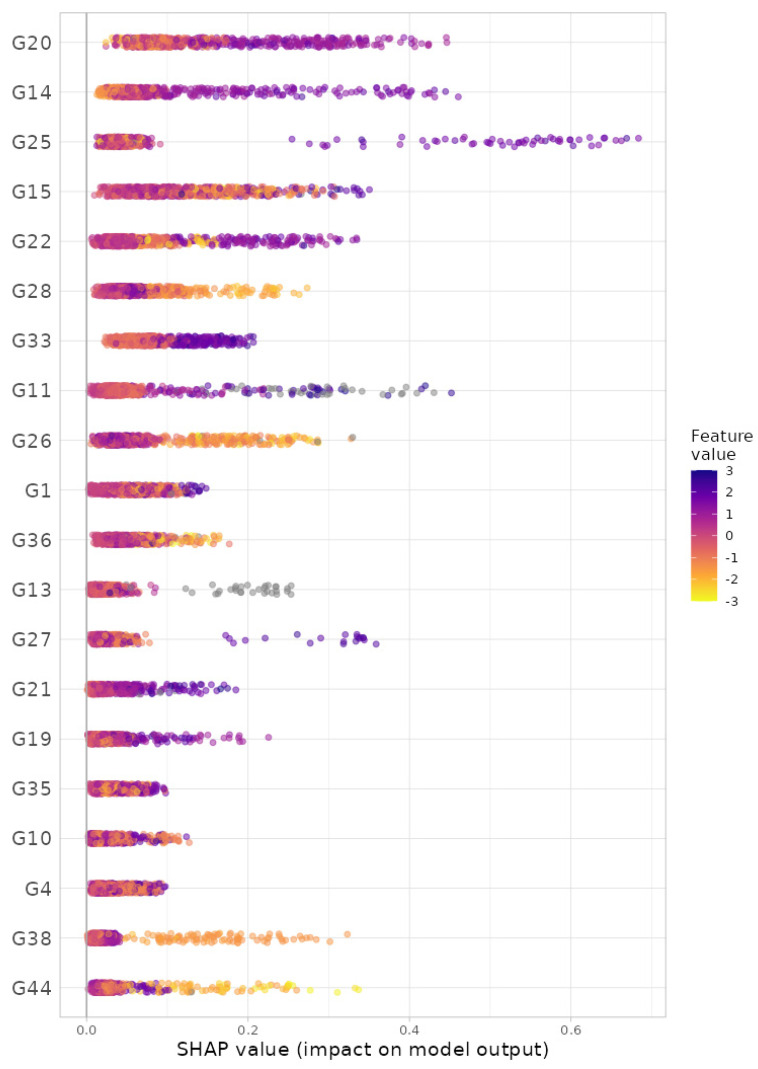
The SHAP variable importance plot at the genus level from gradient boosting algorithm. G20: *Roseburia*; G14: *SMB53*; G25: *Lachnospira*; G15: *Oscillospira*; G22: *Dorea*; G28: *Akkermansia*; G33: *Paraprevotella*; G11: *Acidaminococcus*; G26: *Bifidobacterium*; G1: *Streptococcus*; G36: *Parabacteroides*; G13: *Sarcina*; G27: *Collinsella*; G21: *Ruminococcus*; G19: *Blautia*; G35: *Alistipes*; G10: *Phascolarctobacterium*; G4: *Eubacterium*; G38: *Butyricimonas*; G44: *Sutterella*.

**Figure 7:**
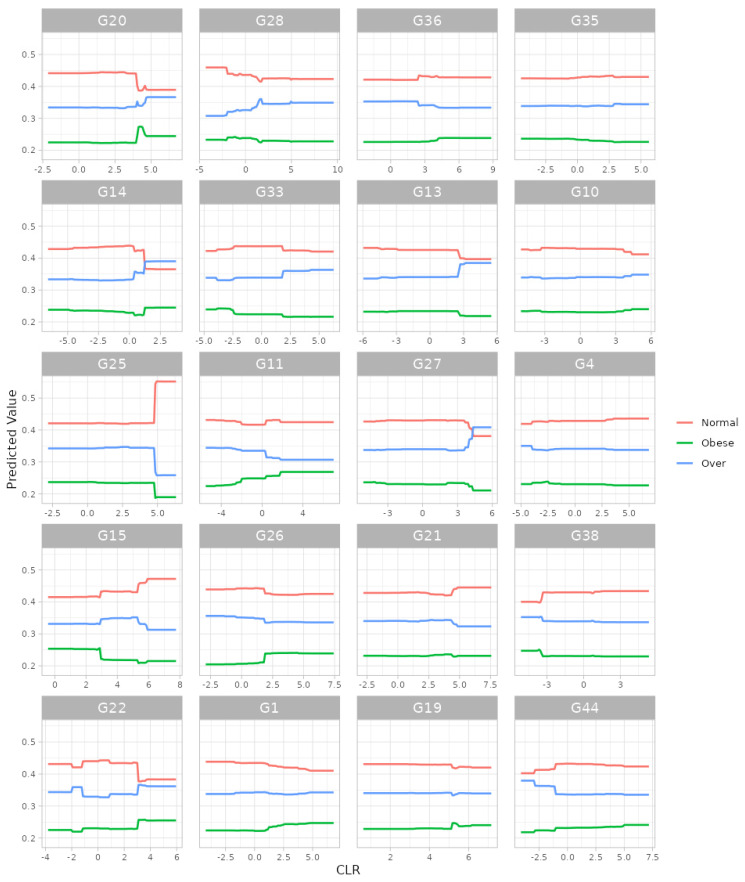
The partial dependence plot at the genus level from gradient boosting algorithm. G20: *Roseburia*; G14: *SMB53*; G25: *Lachnospira*; G15: *Oscillospira*; G22: *Dorea*; G28: *Akkermansia*; G33: *Paraprevotella*; G11: *Acidaminococcus*; G26: *Bifidobacterium*; G1: *Streptococcus*; G36: *Parabacteroides*; G13: *Sarcina*; G27: *Collinsella*; G21: *Ruminococcus*; G19: *Blautia*; G35: *Alistipes*; G10: *Phascolarctobacterium*; G4: *Eubacterium*; G38: *Butyricimonas*; G44: *Sutterella*.

For the gradient boosting algorithm [33,34], we selected (i) 5-fold cross-validation, (ii) the maximum number of trees in the boosting process as 10,000, (iii) the learning rate as 0.001, (iv) the number of taxa to be displayed as 20 and (v) taxonomic ranks to be surveyed as ‘from phylum to genus (16S)’. Here, we selected the maximum training capacities (i.e., the maximum number of trees: 10,000; the learning rate: 0.001) since we intended to gladly endure some slow computation to obtain better results. Then, we found, at the genus level, G20: *Roseburia*; G14: *SMB53*; G25: *Lachnospira*; G15: *Oscillospira*; G22: *Dorea*; G28: *Akkermansia*; G33: *Paraprevotella*; G11: *Acidaminococcus*; G26: *Bifidobacterium*; G1: *Streptococcus*; G36: *Parabacteroides*; G13: *Sarcina*; G27: *Collinsella*; G21: *Ruminococcus*; G19: *Blautia*; G35: *Alistipes*; G10: *Phascolarctobacterium*; G4: *Eubacterium*; G38: *Butyricimonas*; and G44: *Sutterella* as the top 20 most important microbial taxa in predicting levels of BMI (Figure 6). We can also see their relative abundances are larger for lower levels of BMI (Figure 7). Thus, these genera might be beneficial microbes in preventing obesity. Again, in a clinical sense, we might be able to lower obesity levels by increasing their relative abundances using dietary supplements or therapeutics.

## 4. Discussion

In this paper, we introduced a cloud platform, MiMultiCat, for the analysis of microbiome data with multi-categorical responses. We summarize the two main features of MiMultiCat as follows: (i) it is user-friendly, streamlining a long sequence of microbiome data preprocessing and analytic procedures on a step-by-step web environment, and (ii) it is comprehensive, performing both association testing and prediction modeling extensively. We also demonstrated the use of MiMultiCat through the reanalysis of gut microbiome data on obesity with BMI categories [35].

The field of the human microbiome is rapidly emerging, and many people from various disciplines (e.g., biology, medicine, public health) conduct human microbiome studies to discover novel microbial biomarkers that are important in human health or disease. However, there are many different preprocessing and analytic procedures that are involved in the analysis of microbiome data; hence, it is demanding, time-consuming and laborious. Yet, we stress again that MiMultiCat is easy to use and, thus, can benefit many human microbiome researchers as a well-planned routine software for the analysis of microbiome data with multi-categorical responses.

However, we note that association or prediction does not necessarily imply causation. To discover causal microbial biomarkers, investigators need to study further the key underlying mechanisms, such as immunologic or metabolic regulations and digestive processes, of the human microbiome. In addition, scientific knowledge, experimental design, bioengineering technology, bioinformatic or statistical methodology and so forth all together can aid in human microbiome research. Yet, we could not achieve all such goals in this research.

MiMultiCat is freely available on our web server (http://mimulticat.micloud.kr). When the server is busy, users can alternatively run it using their local computer through our GitHub repository (https://github.com/jkim209/mimulticatgit). We monitor our web server and GitHub repository periodically. We are committed to maintaining MiMultiCat at the highest quality. If you have any question or problem using MiMultiCat, you can report it on our GitHub page (https://github.com/jkim209/mimulticatgit/issues) or email the maintainer, Jihun Kim (toujours209@gmail.com).

## Figures and Tables

**Table 1:** The statistical methods available for alpha diversity analysis and taxonomic analysis.

	Nominal Variable	Ordinal Variable
Covariate-adjusted analysis	Multinomial logistic regression	Proportional odds model
Unadjusted analysis	ANOVA F-test and Tukey’s test, Kruskal–Wallis and Dunn’s test, Multinomial logistic regression	Proportional odds model

**Table 2:** The statistical methods available for beta diversity analysis.

	Nominal Variable	Ordinal Variable
Covariate-adjusted analysis	MiRKAT-MC	MiRKAT-MC
Unadjusted analysis	PERMANOVA MiRKAT-MC	MiRKAT-MC

## Data Availability

The 16S raw sequence data are publicly available from the European Bioinformatics Institute (EMBL-EBI) database with access numbers ERP006339 and ERP006342 [35]. We processed them using QIIME 1.7.0 [15,16] and FastTree [47] based on the GreenGenes 12.10 database (https://greengenes.secondgenome.com, access date: 1 November 2023) to construct the feature table, taxonomic table and phylogenetic tree. The final processed microbiome data together with the meta/sample data are also available as example data in the Data Input module so that our users can easily comprehend suitable data formats.
